# Technology-enhanced simulation for healthcare professionals: A meta-analysis

**DOI:** 10.3389/fmed.2023.1149048

**Published:** 2023-04-17

**Authors:** Aaron A. Mitchell, Edward R. Ivimey-Cook

**Affiliations:** ^1^School of Health Sciences, Faculty of Biology, Medicine and Health, The University of Manchester, Manchester, United Kingdom; ^2^School of Biodiversity, One Health and Veterinary Medicine, University of Glasgow, Glasgow, United Kingdom

**Keywords:** medical education, clinical assessment and examination, medical students, virtual reality, high-fidelity simulation

## Abstract

**Aim:**

There have been substantial changes in the simulation technology landscape, in particular virtual reality (VR), during the past decade, which have resulted in increased abundance and decreased cost. We therefore updated a previous meta-analysis conducted in 2011, aiming to quantify the impact of digital technology-enhanced simulation (T-ES) compared with traditional teaching in physicians, physicians-in-training, nurses, and nursing students.

**Design:**

We conducted a meta-analysis consisting of randomized controlled trials published in English between January 2011 and December 2021 in peer-reviewed journals indexed in seven databases. Moderators for study duration, instruction, type of healthcare worker, type of simulation, outcome measure, and study quality rated by Medical Education Research Study Quality Instrument (MERSQI) score were included in our model and used to calculate estimated marginal means (EMMs).

**Results:**

The overall effect of T-ES was positive across the 59 studies included in the analysis compared with traditional teaching [overall effect size 0.80 (95% CI 0.60, 1.00)]. This indicates that T-ES is effective in improving outcomes across a wide variety of settings and participants. The impact of T-ES was found to be greatest for expert-rated product metrics such as procedural success, and process metrics such as efficiency, compared with knowledge and procedure time metrics.

**Conclusions:**

The impacts of T-ES training on the outcome measures included in our study were greatest in nurses, nursing students and resident physicians. T-ES was strongest in studies featuring physical high-fidelity mannequins or centers, compared with VR sensory environment T-ES, though there was considerable uncertainty in all statistical analyses. Further high-quality studies are required to assess direct effects of simulation training on patient and public health outcomes.

## Introduction

Technology-enhanced simulation (T-ES) allows learners to develop their knowledge and skills without exposing real patients to potential harm. T-ES enables clinicians to train for low-frequency, high-intensity events where other forms of medical education may not provide a sufficiently realistic experience. T-ES also allows healthcare professionals to gain insight into clinical practice from multiple perspectives, including that of the patient. For example, a nationwide study in the United States concluded that high-quality simulation experiences could substitute up to 50% of traditional clinical hours across the prelicensure nursing curriculum (1). This is, however, dependent on simulation exercises being of sufficient quality, and it is therefore important for educators to have strong supporting evidence for increased adoption of simulation.

There are numerous potential benefits to the adoption of T-ES: simulation could contribute to increasing both quality and capacity of healthcare training, provided it is sufficiently realistic with opportunities to apply learning, both of which are key themes when frontline healthcare professionals are surveyed (2). Aside from this, the use of high-fidelity mannequins has been shown to encourage the development of professional identity among nurses (3). Also, newly qualified physicians entering the hospital environment often report feeling scared and underprepared when facing acutely ill, predominantly elderly patients (4). Simulation has been demonstrated to be effective in increasing medical students' confidence when entering practice (5), and has been demonstrated to be an effective approach for reducing anxiety and increasing self-confidence, compared with conventional didactic teaching (6). However, a compromise must be made between realism and cost, whilst maintaining a level of realism appropriate to each stage of clinicians' professional development. High-fidelity simulation centers featuring realistic hospital ward or surgical environments require substantial resources alongside the clinical team: static, specialized technology, technicians, actors and psychologists, as well as debriefing and human factors experts (7). Therefore, the advantages of higher-fidelity simulation must be evidence-based, especially as low-fidelity simulation modalities that do not feature digital technology, such as bench-top models or standardized patients, can be a lower-cost and less resource-intensive alternative. In particular, in the past decade, virtual reality (VR) technology has become more attractive for educators due to a substantial decrease in headset cost, with a simultaneous increase in availability due to the rise of low-cost smartphone-based VR headsets, which have combined to make T-ES more accessible globally (8).

The main finding of the Cook et al. (9) study was that T-ES was “consistently associated with large effects for outcomes of knowledge, skills, and behaviors and moderate effects for patient-related outcomes.” However, this analysis focused predominantly on *quasi*-experimental studies or randomized studies with no intervention control groups.

Today, the technology landscape is markedly different than what existed when Cook et al. (9) was conducted, as both VR and smartphone technology was still relatively novel at that time, and potentially beyond the means of medical educators particularly in low-to-middle-income countries. We aimed to update the results of Cook et al. (9) in light of the latest simulation technology and novel research. We also used more focused search criteria and improved statistical methodology, focusing on studies with traditional education control groups and using estimated marginal means (EMMs) to assess the efficacy of simulation across subgroups within the included studies. Here, we provide an evidence-based assessment of T-ES compared with traditional training using a meta-analysis framework. Our analysis is especially important as few reviews published since Cook et al. (9) have focused on simulation in physicians practicing non-surgical specialties.

## Methods

The Preferred Reporting Items for Systematic Reviews and Meta-Analyses (PRISMA) (10) guidance was followed throughout this meta-analysis. Our inclusion criteria followed the PICOS (Population, Intervention, Comparison, Outcomes and Study design) framework. Included participants were physicians, physicians-in-training, medical students, nurses or nursing students. Interventions were any type of digital high-fidelity mannequin or simulation center, VR, defined as exploration and manipulation of computer-generated three-dimensional (3D) sensory environments; and virtual patient (VP) simulation, where learners interact with computerized patient cases and scenarios. We excluded low- or mid-fidelity studies, those featuring real-life standardized patients, and computer-based e-learning studies, which were beyond our definition of T-ES as we aimed to incorporate the latest interactive digital technology. The comparator was equivalent traditional teaching on the same topic. Outcome measures were clinical skills or procedural quality assessed by expert raters, patient health outcomes, knowledge assessed by written examinations, and time metrics. To meet our inclusion criteria, post-tests conducted immediately after either T-ES or didactic training were not conducted in T-ES settings, in order to assess transfer of skills.

We based our search strategy on Cook et al. (9), and made minor alterations as advised by an academic librarian. Detailed search terms are provided in Supplementary Appendix 1. A filter for studies published between January 2011 and December 2021 was applied to all searches, to incorporate those published in decade following Cook et al. (9).

**Figure 1:**
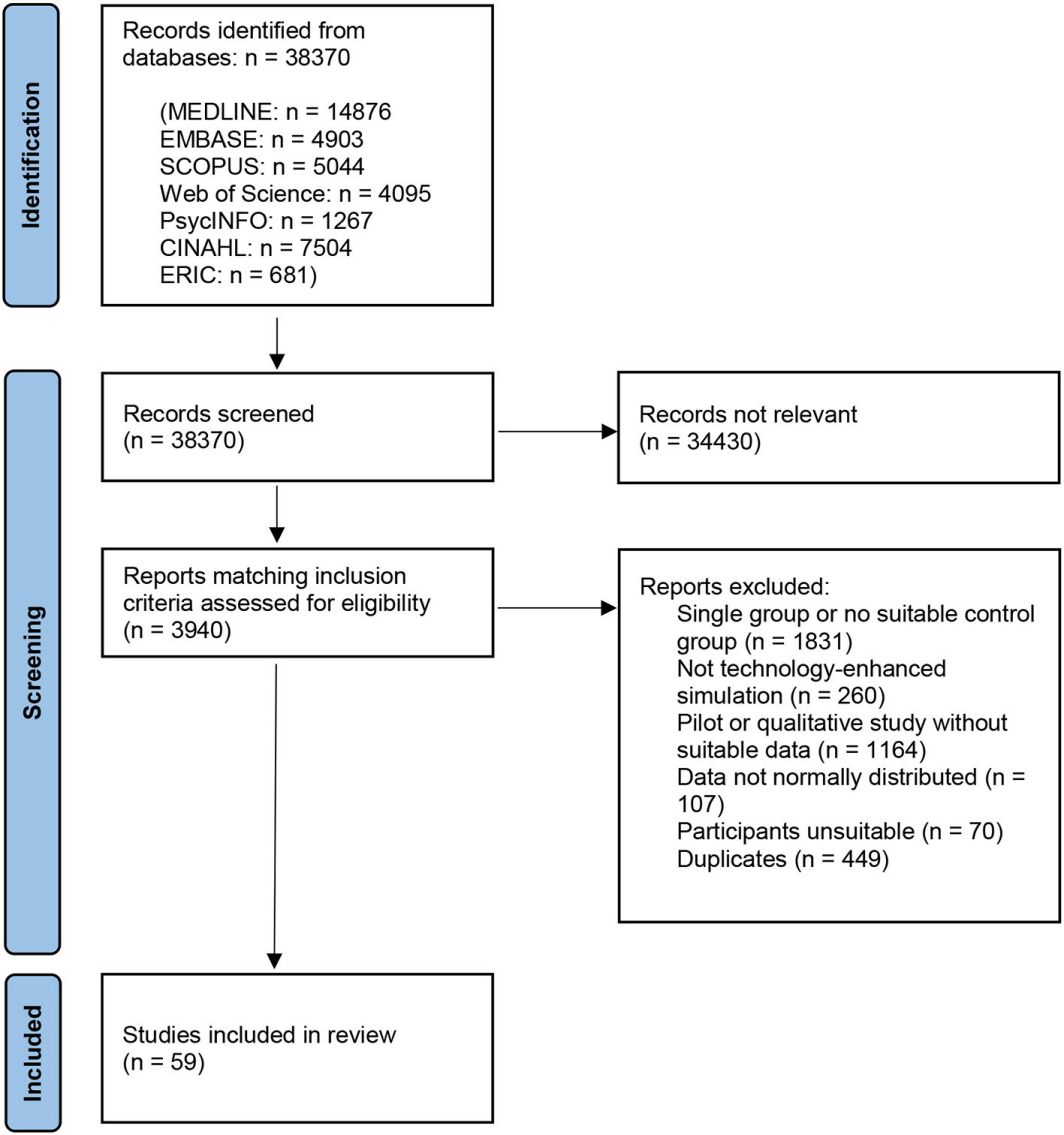
PRISMA flow chart—of 38,370 studies screened, 3,940 abstracts were searched in detail, of which 449 were duplicates–59 studies were included overall, with 136 individual effect sizes analyzed.

We performed a search of seven literature databases: MEDLINE (*n* results = 14,876), Embase (*n* = 4,903), Scopus (*n* = 5,044), Web of Science (*n* = 4,095), PsycINFO (*n* = 1,267), CINAHL (*n* = 7,504), and ERIC (*n* = 681), which was conducted in April and May 2022 (Figure 1), with the last search carried out on May 31st, 2022. Reference lists of all studies meeting the inclusion criteria were searched, however no additional suitable studies were identified. Potentially relevant studies were recorded and once all searches were complete, studies were assessed against eligibility criteria. Post-test data were then extracted from intervention and control groups of suitable studies and added to a pre-prepared spreadsheet. All suitable outcome measures (expert ratings of process or product measures, patient outcomes, time or knowledge scores) reported as a mean value with standard deviation (SD), or information that could be used to calculate SD, were extracted from each paper. Any papers which did not contain suitable data for calculating standardized mean differences (SMDs)—for example, only providing *p-*values or median data—were excluded. Data analysis was conducted using a random-effects multilevel meta-analysis method, in order to account for multiple non-independent effect sizes within each study, for example multiple expert ratings of different aspects of the same procedure. The metafor (11) 3.9 package for R (12) 4.2.2 was used for meta-analysis, with the orchaRd (13) 2.0 package used for data visualization. Emmeans (14) 1.8.2 was used to calculate EMMs. dmetar (15) 0.0.9 was used to calculate heterogeneity across the multilevel model. An overall effect size was calculated using all outcome measures, followed by moderator and EMM analyses. R script used for the meta-analysis can be found in Supplementary Appendix 3.

We excluded *quasi*-experimental studies as scores at baseline and post-test are not independent, and many single-group pre-test/post-test studies do not report correlation values, which are required to calculate SMDs. Between-group SMDs also better account for confounding effects due to individual differences between participants (16). We also did not pool SMDs sizes using subgroup analyses as these can be liable to various statistical and methodological issues such as a failure to reflect uneven sample sizes, spurious correlations with other variables and inflated type-I errors (17). We instead used moderator analysis and EMMs to evaluate the included studies. Moderators for study duration, instruction, type of healthcare worker, type of simulation, outcome measure and study quality rated by Medical Education Research Study Quality Instrument (MERSQI) score were included in our model and used to calculate EMMs.

All studies were appraised using the MERSQI (18), a validated instrument which has been demonstrated to be reliable in similar studies (19)—this was used to examine whether there was any statistically significant relationship between study quality and effect size. Publication bias, time-lag bias (when the results of negative trials take substantially longer to publish than positive trials), and small study bias tests were performed by fitting mean-centered year and effective sample size as moderators using a meta-regression method suitable for high-heterogeneity multilevel meta-analyses (20). Funnel plots were not used due to subjectivity, whilst the trim-and-fill method was not used as its performance is especially poor when there is between-study heterogeneity and no publication bias (21).

## Results

Overall, 59 studies met our inclusion criteria and were included in the meta-analysis, which produced 136 individual effect sizes (Supplementary Figure 1 and Supplementary Appendix 2). We found the mean SMD was 0.80 [(95% CI 0.60, 1.00), *I*^2^ = 84.6%, *p* < 0.0001]—an effect size 0.8 is considered “large”. The mean of the T-ES group is at the 79th percentile of the didactic group, therefore a participant from the T-ES group with a mean score for that group would obtain a higher score than 79% of the participants from the didactic group (22). Details of individual study methodologies can be found in Supplementary Table 1. The mean Medical Education Research Study Quality Instrument (MERSQI) score was 12.8 (±2.48) out of a possible 18, indicating that study quality was generally high.

Total heterogeneity was considerable (*I*^2^ = 84.6%), of which 52.6% was due to between-study heterogeneity and 32% was from heterogeneity between the individual effect sizes. A moderator test for publication bias and time-lag bias was conducted, which was not statistically significant [estimated effect size −0.10 (95% CI −0.27, 0.065), *p* = 0.23; −0.028 (−0.10, 0.045), *p* = 0.45]. Moderator analysis also confirmed that there was no statistically significant impact of study quality (MERSQI score) on the overall effect size [−0.11 (−0.26, 0.031), *p* = 0.12], which means that lower-quality studies did not contribute to an unduly strong effect size.

The impact of T-ES across our included outcome measures was found to be greatest in nurses and nursing students [*n* = 20, EMM 1.11 (0.55, 1.67)] and residents [*n* = 59, 0.92 (0.57, 1.27)], and smallest in medical students [*n* = 44, 0.55 (0.11, 1.00)] and physicians in practice [*n* = 13, 0.64 (-0.16, 1.44)]. High-fidelity mannequins or physical environments [*n* = 52, 0.90 (0.51, 1.28)] were then compared to studies featuring simulated VR environments [*n* = 77, 0.70 (0.36, 1.03)]. VP case-based T-ES demonstrated the greatest EMMs of any modality [*n* = 7, 1.28 (0.46, 2.10)], though this result should be interpreted with caution due to a small sample size and below-average MERSQI scores (range 9.5–11.5). Lastly, average scores were greater when T-ES training was carried out over more than 1 day [0.94 (0.63, 1.24)], compared with single-day exercises [0.58 (0.19, 0.96)], which may suggest a dose-response relationship where a greater duration of T-ES training leads to greater scores in our included outcome measures.

## Discussion

This meta-analysis included a diverse range of relevant outcome measures over three different high-fidelity simulation modalities—high-fidelity mannequins and centers, VR and VPs—and physicians and nurses at all stages of training. The overall effect size of 0.80 [0.60, 1.00] shows a strong immediate effect of T-ES training when translated to knowledge scores, clinical settings involving real patients, and dexterous surgical tasks involving cadaver or porcine models. These results support the consensus that high-fidelity simulation is best suited to refining performance as opposed to knowledge, with the largest effects shown for process and product outcomes as opposed to time or knowledge. Broadly, this suggests that T-ES can contribute to improving patient care and prepare healthcare trainees for unfamiliar situations.

In comparison with the Cook et al. (9) study, we found an EMM of 0.55 [0.16, 0.94] for knowledge outcomes (written examination assessments on the topic of interest), compared with a pooled effect size of 1.20 [1.04, 1.35]. Scores for expert-rated outcomes assessed in real patient cases, cadaver or animal models were notably similar: 1.16 [0.84, 1.48], compared with 1.18 [0.98, 1.37] in the Cook et al. (9) analysis. The EMM for process metrics was 0.92 [0.52, 1.33] compared with a pooled effect size of 1.09 [1.03, 1.16], and 0.57 [0.16, 0.99] compared with 1.14 [1.03, 1.25] for time skills. It is notable that these effects were similar despite methodological differences, principally that we evaluated studies with didactic teaching control groups, as opposed to the no intervention control groups evaluated in the Cook et al. (9) analysis, something that has been a criticism of previous analyses (23). However, as no overall effect size was provided in the Cook et al. (9) analysis, we are unable to make a direct comparison, but we can state that T-ES remains relevant in achieving desired learning outcomes despite the increasing abundance of technology in the past decade.

Evidence has suggested that low-fidelity simulation is most impactful in building knowledge, whilst higher-fidelity simulation is best used to develop performance and action (24). It is important to note that one recent economic analysis found that VR training required 22% less time than more traditional high-fidelity simulation to achieve the same learning outcomes, at a 40% lower cost (25). This must be considered in the context of evidence that when trainees are exposed to high-fidelity simulation at an early stage, this may lead to overconfidence relative to lower-fidelity interventions (26). This is supported by the results of another recent meta-analysis (27) of 8 VR studies, which found only a medium effect size for knowledge outcomes [0.44 (0.18, 0.69)]. Interestingly, we also found that the average effect of T-ES was greater when no instruction was given simultaneously by clinical academics [EMM 1.13 (0.71, 1.55)], compared to when instruction was given [0.60 (0.31, 0.89)]. However, as this study only assessed immediate post-test results, we cannot determine if this is a longer-term effect on retention following simulation training. In the existing literature, a progressive curriculum from low-fidelity to high-fidelity simulation has been shown to be effective (28), where more inexperienced students learn with minimal extraneous stimuli until reaching proficiency, preventing cognitive overload. This is also something we lacked the statistical power to examine.

Due to limited randomized studies with suitable control groups, we lacked the statistical power to make definitive comparisons between types of simulation or healthcare professionals using EMMs. This was apparent when examining a difference in outcomes of T-ES between physicians at each stage of training, or between physicians and nurses, as almost three-quarters of included studies evaluated physicians-in-training, it was difficult to draw statistically powerful conclusions, something that is also highlighted by a relative lack of reviews evaluating simulation in practicing physicians.

## Conclusion

We used a broad search strategy to synthesize 59 generally high-quality randomized studies to contribute to the evidence base for simulation in diverse healthcare settings.

We found that skills and knowledge developed during T-ES are generally transferable to other settings across physicians and nurses at all stages of training, types of high-fidelity simulation modality and numerous clinical specialties. However, this must be considered with considerable uncertainty and heterogeneity taken into account. Differing inclusion criteria, study designs and statistical methodology mean that fair and direct comparisons with Cook et al. (9) are difficult.

With a tendency to focus on novel, rapidly advancing technologies such as VR, existing simulation modalities such as low-fidelity bench-top models or simulated patients may perhaps not receive equal consideration. Lower-fidelity methods can still achieve the desired learning outcomes, especially in more inexperienced trainees. There remains a clear need to identify whether simulation is effective in improving the quality and scalability of medical education, and care delivered to patients, relative to lower-fidelity, less expensive interventions.

## Data availability statement

The original contributions presented in the study are included in the article/Supplementary material, further inquiries can be directed to the corresponding author.

## Author contributions

AM devised the study concept, conducted the literature searches, and drafted the first version of the manuscript. AM and EI-C conducted the meta-analysis and redrafted the manuscript. Both authors contributed to the article and approved the submitted version.
