# Supplementary material for: Technology-enhanced simulation for healthcare professionals: A meta-analysis
Source: Front Med (Lausanne). 2023 Apr 17;10:1149048. doi: 10.3389/fmed.2023.1149048 (PMC10150956; doi:10.3389/fmed.2023.1149048)
Supplement: Supplementary file 1 [file Data_Sheet_1.docx]

**Supplemental Material**

**Contents:**

Supplementary Table 1

Supplementary Figure 1

Supplementary Appendix 1 (Search terms)

Supplementary Appendix 2 (Studies included in meta-analysis)

Supplementary Appendix 3 (R script)

| **Study name** | **Population and study duration** | **Intervention** | **Control** | **Tests or outcomes** |
| --- | --- | --- | --- | --- |
| Aloush, 2018 | Baccalaureate student nurses, single institution (1 month) | 3 sessions of 2 hours each on Laerdal IV Torso with expert demonstration | 3 sessions of 2 hours each, PowerPoint lecture and video | 23-item MCQ pre-test and post-test, unblinded |
| Andreasen et al., 2019 | Physicians (obstetric trainees and experienced consultants), six obstetric departments (21 months) | Medaphor Scantrainer transabdominal ultrasound VR with instruction and feedback until mastery reached | Traditional clinical placement | Expert-rated blinded image quality scores on real patients, 2 blinded raters |
| Bloch et al., 2017 | ICU trainees, single institution (1 day) | 4 hours free practice using a TEE (HeartWorks) mannequin simulator | 4 hours of lecture-based training | Expert-rated blinded TEEs on real patients, 2 blinded raters |
| Boscolo-Berto et al., 2021 | 2^nd^ year medical students, single institution (1 week) | The Anatomage Table VR free practice dissection + gross dissection | Textbook study + gross dissection | Written examination pre-test and post-test, unblinded |
| Cannon et al., 2014 | 3^rd^ year orthopaedic residents, multicentre (5.5 months) | ArthroSim VR to proficiency level based on average proficiency of 5 surgeons | Traditional training | Expert-rated blinded diagnostic knee arthroscopy on real patients, 10 blinded expert raters |
| Chao et al., 2021 | Nursing students, single institution (9 months) | 10-20 minute interactive VR course | Video instruction | Written examination pre-test and post-test, unblinded |
| Cortegiani et al., 2015 | Medical students, single institution (2 months) | Traditional lectures + one day instructor-led SimMan 2 course with debriefing + critical appraisal | Traditional lectures | Written examination pre-test and post-test, blinded |
| da Cruz et al., 2016 | Medical students, single institution (1 day) | VR warm-up + porcine model procedure | Porcine model procedure | Expert-rated blinded laparoscopic cholecystectomy on porcine model, 2 blinded expert raters |
| Desender et al., 2016 | Surgeons, multicentre (21 months) | Patient-specific VR rehearsal | Standard procedure | Randomised patient list, real patient health outcomes, unblinded |
| Diehl et al., 2017 | Physicians, multicentre (1 day) | 4-hour VP activity | Lectures and case discussion | Written examination pre-test and post-test, unblinded |
| Ekstrand et al., 2018 | Medical students, single institution (1 day) | 17-minute VR activity with instruction | Traditional paper-based study | MCQ pre-test and post-test, blinded |
| Etienne et al., 2021 | Medical students, multicentre (30 months) | 3-month traditional placement + 2-hour Medaphor course with instruction | 3-month traditional placement | Pre-test and post-test on real patient, blinded |
| Fernandez et al., 2020 | Residents, single institution (20 months) | 1-hour didactic training + 3-hour series of instructor-led high-fidelity simulations with debriefing | Traditional orientation | Expert-rated resuscitation performance on real patients, blinded |
| Ferrero et al., 2014 | Residents, single institution (1 week) | 45-minute didactic training + Heartworks TEE mannequin with guidance + free practice | 45-minute didactic PowerPoint + video | Blinded expert-rated image scores on real patients, 3 blinded raters |
| Fischer et al., 2018 | Medical students, single institution (1 day) | Instructor-led Mentice VIST-Lab CA simulator course + free practice | Didactic PowerPoint | MCQ post-test, blinded |
| Franzeck et al., 2012 | Medical students, single institution (3 weeks) | 3-week haptic VR training, expert-led with critical appraisal | Traditional placement and assistance with surgery | Pre-test and post-test OSCE, 5 blinded raters |
| Gu et al., 2017 | 2^nd^ year nursing students, single institution (4 months) | Traditional didactic + 30-minute VP activity | Traditional didactic | Written examination post-test, unblinded |
| Guerrero et al., 2021 | Nursing students, single institution (1 year) | Traditional placement + various high-fidelity simulated scenarios with debriefing | Traditional placement | Expert ratings on real patients, blinded and written examination post-test |
| Hooper et al., 2019 | Residents, single institution (1 day) | 2 VR simulation practice attempts | Standard study materials | Cadaver pre-test and post-test, blinded, and written examination post-test |
| Hou et al., 2018 | Residents, single institution (1 day) | Haptic VR free practice | Traditional didactic | Cadaver image rating post-test, unblinded |
| Jokinen et al., 2019 | Residents, multicentre (3.5 years) | E-learning + LAP Mentor VR simulator practice over a 1-month period | Standard clinical education | Blinded expert-rated surgical performance on real patients, 3 blinded raters |
| Kerr et al., 2013 | Interns and residents, multicentre (1 day) | Instructor-led 30-minute Noelle mannequin session, 15-minute debriefing and 10-minute didactic session | 55-minute case-based interactive workshop | MCQ pre-test and post-test, unblinded |
| Konge et al., 2015 | Physicians, multicentre and international (1 day) | Half-day supervised training on GI Bronch Mentor simulator | Standard observation of procedures | Blinded expert-rated procedures on real patients, 3 blinded raters |
| Lebdai et al., 2021 | 4^th^ year medical students, single institution (3 months) | 3 90-minute VP cases with debriefing | 12 hours traditional training | Written examination post-test (prior to crossover), unblinded but questions set by blinded researcher |
| Logishetty et al., 2019 | Surgical trainees, multicentre (6 weeks) | Procedure demonstration + video, VR demonstration + weekly structured training with 45-60 minute sessions | Procedure demonstration + video, traditional training | Expert-rated cadaver post-test, blinded |
| Lohre et al., 2020a | Residents, multicentre (3 days) | IVR training until programme-defined proficiency reached, unlimited free practice | Unlimited video instruction | Blinded expert-rated cadaver and written examination and post-test |
| Lohre et al., 2020b | Residents and surgeons, multicentre (1 day) | IVR training until programme-defined proficiency reached, unlimited free practice | Unlimited reading of a journal article | Expert-rated cadaver and written examination post-test, 10 blinded raters |
| Maddry et al., 2014 | Residents, single institution (1 day) | 4 instructor-led SimMan patient cases with debriefing | 20-minute lecture | Written examination pre-test and post-test, unblinded |
| Maertens et al., 2017 | Trainees, multicentre (14 months) | Standard practice + VR proficiency-based endovascular curriculum | Standard practice | Expert-rated procedures on real patients, blinded |
| Mansoory et al., 2021 | Interns, single institution (4 months) | VR-based exercise | 30-minute lecture | MCQ pre-test and post-test, unblinded |
| Matsumura et al., 2018 | 5^th^ year medical students, single institution (2 years) | Standard training + didactic lecture, 45-minute VP exercise | Standard training + didactic lecture | Written examination pre-test and post-test, unblinded |
| McIntosh et al., 2014 | Residents, single institution (4 weeks) | Instructor demonstration on Simbionix GI Mentor II, 16 hours training and 10-20 hours free practice | Traditional demonstration | Blinded expert-rated procedures on real patients, 2 blinded raters |
| Meng et al., 2020 | Trainees, single institution (3 years) | Traditional training + 20 hours instructor-led EMS ERCP simulator training | Traditional training | Expert-rated procedures on real patients, 1 blinded rater |
| Merriman et al., 2014 | 1^st^ year nursing students, single institution (1 day) | Demonstration followed by free practice on high-fidelity mannequin | 1-hour didactic lecture | Pre-test and post-test OSCE, blinded |
| Pietersen et al., 2021 | Physicians, multicentre (6 months) | Didactic + unsupervised VR training | Didactic study (audio and text) | MCQ pre-test, post-test expert rating on real patients, 1 blinded rater |
| Qi et al., 2021 | Residents, single institution (1 day) | 40-minute VR exercise | Didactic lecture and case-based discussion | Written examination pre-test and post-test, unblinded |
| Rebolledo et al., 2015 | Residents, single institution (1 day) | 2.5 hours training on Insight Arthro VR | 2-hour didactic PowerPoint with demonstration | Expert-rated cadaver post-test, 2 blinded raters |
| Ros et al., 2020 | 4^th^ year medical students, single institution (1 day) | Reading + Immersive VR | Reading | MCQ post-test, unblinded |
| Sari et al., 2018 | 3^rd^ year nursing students, single institution (1 day) | 2 15-20 minute simulated scenarios with high-fidelity mannequins and debriefing | Traditional training | Observation with real patient, blinded and written examination pre-test and post-test |
| Schroedl et al., 2012 | 1^st^ year residents, single institution (4 weeks) | 2-hour didactic session followed by 2-hour interactive high-fidelity mannequin session | Traditional training | Expert-rated assessment of real patient care post-test, unblinded but second blinded rater evaluated a 40% random sample to assess reliability |
| Smelt et al., 2015 | Trainees, single institution (1 day) | Free practice on Heartworks simulator | Traditional training | MCQ pre-test and post-test, unblinded but test designed by blinded researcher |
| Smelt et al., 2016 | Trainees, single institution (1 day) | 1-hour taught session on Orpheus simulator with debriefing | 1-hour didactic lecture | MCQ pre-test and post-test, unblinded but test designed by blinded researcher |
| Solymos et al., 2015 | Medical students, single institution (6 weeks) | Instructor-led interactive high-fidelity session | Didactic lectures | MCQ pre-test and post-test, unblinded |
| Starodub et al., 2020 | Nurses, multicentre (1 day) | Introductory lecture + instructor led SimMan session with debriefing | Introductory lecture + case-based lecture | Written examination pre-test and post-test, unblinded |
| Stayt et al., 2015 | 1^st^ year nursing students, multicentre (1 day) | 2-hour instructor-led simulation on ALS simulator | 1-hour didactic lecture | Pre-test and post-test OSCE, blinded |
| Stepan et al., 2017 | Medical students, single institution (1 day) | 10-minute didactic introductory lecture, 5-minute VR introduction, 5-minute VR free practice, 10 minutes text study | 10-minute didactic introductory lecture, 20 minutes text study | Written examination pre-test and post-test, unblinded |
| Subramanian et al., 2012 | 3^rd^ year medical students, single institution (6 months) | 1-hour to practice with 2 VP cases | 2 30-minute didactic PowerPoint lectures | Written examination pre-test and post-test, unblinded |
| Succar et al., 2013 | Medical students, single institution (10 days) | Traditional training and VP-based course | Traditional training | Written examination pre-test and post-test, unblinded |
| Tawalbeh, 2020 | Nursing students, single institution (3 months) | Traditional training, didactic lectures + 9 high-fidelity scenarios with debriefing | Traditional training + didactic lectures | Written examination pre-test and post-test, unblinded |
| Tawfik et al., 2020 | 3^rd^ year medical students, single institution (1 day) | 4 10-minute cases + instructor-led interactive high-fidelity simulation with debriefing | 50-minute didactic lecture with videos | Written examination pre-test and post-test, unblinded |
| Tiffen et al., 2011 | Nursing students, single institution (1 day) | Didactic lecture and demonstration + 1-hour instructor-led high-fidelity session | Didactic lecture + demonstration | Written examination pre-test and post-test, unblinded |
| Tolsgaard et al., 2015 | Residents, multicentre (2 months) | 1-hour didactic lecture, traditional training, VR training until proficiency reached + high-fidelity mannequin practice | 1-hour didactic lecture + traditional training | Expert-rated procedures on real patients, 2 blinded raters |
| Tubaishat and Tawalbeh, 2015 | Nursing students, single institution (2 days) | Various 20-minute instructor-led high-fidelity scenarios with 10-minute debriefing | 2-hour didactic presentation + discussion | Written examination pre-test and post-test, unblinded |
| Urdiales et al., 2020 | Medical students, single institution (1 day) | Demonstration + interactive session with Kelly mannequin | Didactic lectures | Written examination post-test, unblinded |
| Wong et al., 2019 | Medical students and residents, single institution (1 week) | Didactic training, 1-hour ORSIM VR | Didactic teaching | Pre-test and post-test on real patient, 2 blinded expert raters |
| Wu and Beyea, 2017 | Medical students, single institution (1 day) | 30-minute free practice on OtoSim | Traditional teaching with discussion | Written examination pre-test and post-test, unblinded |
| Wu et al., 2018 | Medical students, single institution (1 day) | 30-minute free practice on OtoSim | Traditional teaching with discussion | Pre-test and post-test OSCE, 2 blinded expert raters |
| Xin et al., 2019 | Residents, single institution (1 day) | 20-minute training video + 30 minutes immersive VR | 20-minute training video + 30-minute demonstration | Post-test on cadaver, double-blinded |
| Yamamoto et al., 2019 | Residents, single institution (1 week) | 3 1-hour SimMan scenarios with instructor, discussion + debriefing | 1-hour didactic lecture | Written examination pre-test and post-test, unblinded |

**Supplementary Figure 1.** All effect sizes included in our analysis, weighted by precision, with the intercept from initial model run without moderators.

**Supplementary Appendix 1**

Search terms

**MEDLINE**

1. exp education, medical/ or exp education, nursing/ or exp students, health occupations/ or exp students, nursing/ or exp students, medical/

2. exp faculty/ or exp schools/ or educational, professional/ or "internship and residency"/ or ed.fs.

3. 1 or 2

4. exp *surgical procedures, operative/

5. exp *"Diagnostic Techniques and Procedures"/

6. computer simulation/ or simulat*.ti,ab. or manikin*.mp. or mannikin*.mp. or mannequin*.mp. or models, anatomic/ or virtual.mp. or (bench adj2 model*).mp.

7. 3 and 6

8. (4 or 5) and 7

9. 7 or 8

10. (evaluat* or assess* or compar* or impact* or effect* or validat* or improv* or measure* or reliab* or random* or control* or pretest* or chang* or cohort*).mp.

11. 9 and (10 or educational measurement/)

12. 11 and 2011:2021.(sa_year).

**Embase**

1. exp education, medical/ or exp education, nursing/ or exp students, health occupations/ or exp students, nursing/ or exp students, medical/

2. exp faculty/ or exp schools/ or educational, professional/ or "internship and residency"/ or ed.fs.

3. 1 or 2

4. exp *surgical procedures, operative/

5. exp *"Diagnostic Techniques and Procedures"/

6. computer simulation/ or simulat*.ti,ab. or manikin*.mp. or mannikin*.mp. or mannequin*.mp. or models, anatomic/ or virtual.mp. or (bench adj2 model*).mp.

7. 3 and 6

8. (4 or 5) and 7

9. 7 or 8

10. (evaluat* or assess* or compar* or impact* or effect* or validat* or improv* or measure* or reliab* or random* or control* or pretest* or chang* or cohort*).mp.

11. 9 and (10 or educational measurement/)

12. 11 and "controlled study".sa_suba.

13. 12 and 2011:2021.(sa_year).

**PsycINFO**

1. exp graduate education/

2. “Professional Education & Training”.cc.

3. “Curriculum & Programs & Teaching Methods”.cc.

4. exp computer simulation or exp simulation/

5. exp health personnel/

6. (1 or 5) and (2 or 3) and simulation*.mp. [mp = title, abstract, heading word, table of contents, key concepts]

7. (1 or 5) and 4

8. 6 or 7

9. 8 and 2011:2021.(sa_year).

**ERIC**

(healthcare OR medical OR nursing OR nurses OR surgical) AND (simulat* OR virtual OR manikin* OR manniquin OR cadaver*) AND (education OR teach OR teaching OR learn* OR training OR train OR curricul*) AND (competenc* OR skills OR skill OR assessment OR compare OR comparative OR comparison OR measure* OR evaluat*)

Limit to 2011-01-01 – 2021-12-31

**Web of Science**

1. Topic=((medical or nurse* or nursing or clinical or hospital* or physician* or surgical) SAME (educat* or student* OR interns* or train* or residency) AND (simulat* or virtual or cadaver* or manikin* or mannikin* or mannequin* or "sim man" or harvey or laerdal or "vr mist")) AND Topic=(competenc* or skill* or mastery or measure* or compar* or proficien* or quality)

2. TI=((medical or nurse* or nursing or clinical or hospital* or physician* or surgical) SAME (educat* or student* OR interns* or train* or residency))

3. 2 AND 1 Refined by: Document Type=(ARTICLE) Timespan=2011-2021

**Scopus**

( ( TITLE-ABS-KEY ( ( simulat* OR virtual OR manikin* OR mannikin* OR "sim man" OR harvey OR laerdal OR "vr mist" OR cadaver* ) AND ( medical OR surgical OR clinician* OR resident OR physician* OR nurs* OR "health care" ) ) AND TITLE-ABS-KEY ( ( educat* OR train* OR student* ) AND ( performance OR proficien* OR mastery OR skill* OR competen* ) ) ) ) AND TITLE ( simulat* OR virtual OR manikin* OR mannikin* OR "sim man" OR harvey OR laerdal OR "vr mist" OR cadaver* ) AND TITLE-ABS-KEY ( outcome* OR trial* OR program* OR evaluat* OR assess* OR measure* OR compar* ) AND NOT ( "standardized patients" OR "human standardized" ) AND NOT SRCTYPE ( "article in press" ) AND ( LIMIT-TO ( PUBYEAR , 2021 ) OR LIMIT-TO ( PUBYEAR , 2020 ) OR LIMIT-TO ( PUBYEAR , 2019 ) OR LIMIT-TO ( PUBYEAR , 2018 ) OR LIMIT-TO ( PUBYEAR , 2017 ) OR LIMIT-TO ( PUBYEAR , 2016 ) OR LIMIT-TO ( PUBYEAR , 2015 ) OR LIMIT-TO ( PUBYEAR , 2014 ) OR LIMIT-TO ( PUBYEAR , 2013 ) OR LIMIT-TO ( PUBYEAR , 2012 ) OR LIMIT-TO ( PUBYEAR , 2011 ) )

**CINAHL**

S1 (MH "Education, Medical+") or (MH "Education, Nursing+") or (MH "Students, Health Occupations+") or (MH "Students, Nursing+") or (MH "Students, Medical")

S2 (MH "Faculty+") or (MH "Schools+") or “professional educational” or “internship and residency”

S3 S1 or S2

S4 (MH "Surgery, Operative+")

S5 (MH "Diagnosis+")

S6 (MH "Computer Simulation+") or (TI simulat* or AB simulat*) or manikin* or mannikin* or mannequin* or (MH "Models, Anatomic+") or virtual or (bench N2 model*)

S7 S3 and S6

S8 (S4 or S5) and S7

S9 S7 or S8

S10 evaluat* or assess* or compar* or impact* or effect* or validat* or improv* or measure* or reliab* or random* or control* or pretest* or chang* or cohort*

S11 S9 and (S10 or (MH "Educational Measurement+")) Limiters - Publication Year: 2011-2021

Minor variations recommended by the academic librarian were that the Web of Science Full Collection was searched as opposed to only the Science Citation Index Expanded in Cook, and CINAHL search terms were converted from Ovid to EBSCO notation due to a licensing change. Filters for study type used on the original MEDLINE search were removed. The ‘controlled study’ filter was applied to Embase, simplifying a similar filter used in the original search.

**Supplementary Appendix 2**

Data used in this study can be accessed via the Open Science Framework: <https://osf.io/uzcpd/?view_only=6c3506aad9f64ca08015c65153824a14>.

References for studies included in meta-analysis

Aloush, S.M., 2019. Lecture-based education versus simulation in educating student nurses about central line–associated bloodstream infection–prevention guidelines. *Journal of Vascular Nursing*, *37*(2), 125-131.

Andreasen, L.A., Tabor, A., Nørgaard, L.N., Ringsted, C., Sandager, P., Rosthøj, S. and Tolsgaard, M.G., 2019. Is simulation training only for inexperienced trainees? A multicenter randomized trial exploring the effects of simulation-based ultrasound training on obstetricians’ diagnostic accuracy. *Ultrasound in Obstetrics & Gynecology*, *54*, 84-154.

Bloch, A., Von Arx, R., Etter, R., Berger, D., Kaiser, H., Lenz, A. and Merz, T.M., 2017. Impact of simulator-based training in focused transesophageal echocardiography: a randomized controlled trial. *Anesthesia & Analgesia*, *125*(4), 1140-1148.

Boscolo-Berto, R., Tortorella, C., Porzionato, A., Stecco, C., Picardi, E.E.E., Macchi, V. and De Caro, R., 2021. The additional role of virtual to traditional dissection in teaching anatomy: a randomised controlled trial. *Surgical & Radiologic Anatomy*, *43*(4), 469-479.

Cannon, W.D., Garrett Jr, W.E., Hunter, R.E., Sweeney, H.J., Eckhoff, D.G., Nicandri, G.T., Hutchinson, M.R., Johnson, D.D., Bisson, L.J., Bedi, A. and Hill, J.A., 2014. Improving residency training in arthroscopic knee surgery with use of a virtual-reality simulator: a randomized blinded study. *Journal of Bone & Joint Surgery*, *96*(21), 1798-1806.

Chao, Y.C., Hu, S.H., Chiu, H.Y., Huang, P.H., Tsai, H.T. and Chuang, Y.H., 2021. The effects of an immersive 3D interactive video program on improving student nurses' nursing skill competence: a randomized controlled trial study. *Nurse Education Today*, *103*, 104979.

Cortegiani, A., Russotto, V., Montalto, F., Iozzo, P., Palmeri, C., Raineri, S.M. and Giarratano, A., 2015. Effect of high-fidelity simulation on medical students’ knowledge about advanced life support: a randomized study. *PLoS One*, *10*(5), e0125685.

da Cruz, J.A.S., Dos Reis, S.T., Frati, R.M.C., Duarte, R.J., Nguyen, H., Srougi, M. and Passerotti, C.C., 2016. Does warm-up training in a virtual reality simulator improve surgical performance? A prospective randomized analysis. *Journal of Surgical Education*, *73*(6), 974-978.

Desender, L.M., Van Herzeele, I., Lachat, M.L., Rancic, Z., Duchateau, J., Rudarakanchana, N., Bicknell, C.D., Heyligers, J.M., Teijink, J.A. and Vermassen, F.E., 2016. Patient-specific rehearsal before EVAR. *Annals of Surgery*, *264*(5), 703-709.

Diehl, L.A., Souza, R.M., Gordan, P.A., Esteves, R.Z. and Coelho, I.C.M., 2017. InsuOnline, an electronic game for medical education on insulin therapy: a randomized controlled trial with primary care physicians. *Journal of Medical Internet Research*, *19*(3), e6944.

Ekstrand, C., Jamal, A., Nguyen, R., Kudryk, A., Mann, J. and Mendez, I., 2018. Immersive and interactive virtual reality to improve learning and retention of neuroanatomy in medical students: a randomized controlled study. *Canadian Medical Association Journal*, *6*(1), e103-e109.

Etienne, M., Gabay, L., Levaillant, J.M., Vivanti, A., Dommergues, M., Fernandez, H. and Capmas, P., 2021. Benefits of using a simulator in the initial training for transvaginal ultrasound examination in gynecologic emergency unit. *Journal of Gynecology, Obstetrics & Human Reproduction*, *50*(2), 101938.

Fernandez, R., Rosenman, E.D., Olenick, J., Misisco, A., Brolliar, S.M., Chipman, A.K., Vrablik, M.C., Kalynych, C., Arbabi, S., Nichol, G. and Grand, J., 2020. Simulation-based team leadership training improves team leadership during actual trauma resuscitations: a randomized controlled trial. *Critical Care Medicine*, *48*(1), 73-82.

Ferrero, N.A., Bortsov, A.V., Arora, H., Martinelli, S.M., Kolarczyk, L.M., Teeter, E.C., Zvara, D.A. and Kumar, P.A., 2014. Simulator training enhances resident performance in transesophageal echocardiography. *Anesthesiology*, *120*(1), 149-159.

Fischer, Q., Sbissa, Y., Nhan, P., Adjedj, J., Picard, F., Mignon, A. and Varenne, O., 2018. Use of simulator-based teaching to improve medical students’ knowledge and competencies: randomized controlled trial. *Journal of Medical Internet Research*, *20*(9), e9634.

Franzeck, F.M., Rosenthal, R., Muller, M.K., Nocito, A., Wittich, F., Maurus, C., Dindo, D., Clavien, P.A. and Hahnloser, D., 2012. Prospective randomized controlled trial of simulator-based versus traditional in-surgery laparoscopic camera navigation training. *Surgical Endoscopy*, *26*(1), 235-241.

Gu, Y., Zou, Z. and Chen, X., 2017. The effects of vSIM for Nursing as a teaching strategy on fundamentals of nursing education in undergraduates. *Clinical Simulation in Nursing*, *13*(4), 194-197.

Guerrero, J.G., Hafiz, A.H., Eltohamy, N.A.E., Gomma, N. and Al Jarrah, I., 2021. Repeated exposure to high-fidelity simulation and nursing interns’ clinical performance: impact on practice readiness. *Clinical Simulation in Nursing*, *60*, 18-24.

Hooper, J., Tsiridis, E., Feng, J.E., Schwarzkopf, R., Waren, D., Long, W.J., Poultsides, L., Macaulay, W., Papagiannakis, G., Kenanidis, E. and Rodriguez, E.D., 2019. Virtual reality simulation facilitates resident training in total hip arthroplasty: a randomized controlled trial. *The Journal of Arthroplasty*, *34*(10), 2278-2283.

Hou, Y., Shi, J., Lin, Y., Chen, H. and Yuan, W., 2018. Virtual surgery simulation versus traditional approaches in training of residents in cervical pedicle screw placement. *Archives of Orthopaedic & Trauma Surgery*, *138*(6), 777-782.

Jokinen, E., Mikkola, T.S. and Härkki, P., 2019. Effect of structural training on surgical outcomes of residents’ first operative laparoscopy: a randomized controlled trial. *Surgical Endoscopy*, *33*(11), 3688-3695.

Kerr, B., Lee-Ann Hawkins, T., Herman, R., Barnes, S., Kaufmann, S., Fraser, K. and Ma, I.W., 2013. Feasibility of scenario-based simulation training versus traditional workshops in continuing medical education: a randomized controlled trial. *Medical Education Online*, *18*(1), 21312.

Konge, L., Clementsen, P.F., Ringsted, C., Minddal, V., Larsen, K.R. and Annema, J.T., 2015. Simulator training for endobronchial ultrasound: a randomised controlled trial. *European Respiratory Journal*, *46*(4), 1140-1149.

Lebdai, S., Mauget, M., Cousseau, P., Granry, J.C. and Martin, L., 2021. Improving academic performance in medical students using immersive virtual patient simulation: a randomized controlled trial. *Journal of Surgical Education*, *78*(2), 478-484.

Logishetty, K., Rudran, B. and Cobb, J.P., 2019. Virtual reality training improves trainee performance in total hip arthroplasty: a randomized controlled trial. *The Bone & Joint Journal*, *101*(12), 1585-1592.

Lohre, R., Bois, A.J., Athwal, G.S. and Goel, D.P., 2020. Improved complex skill acquisition by immersive virtual reality training: a randomized controlled trial. *Journal of Bone & Joint Surgery*, *102*(6), e26.

Lohre, R., Bois, A.J., Pollock, J.W., Lapner, P., McIlquham, K., Athwal, G.S. and Goel, D.P., 2020. Effectiveness of immersive virtual reality on orthopedic surgical skills and knowledge acquisition among senior surgical residents: a randomized clinical trial. *JAMA Network Open*, *3*(12), e2031217-e2031217.

Maddry, J.K., Varney, S.M., Sessions, D., Heard, K., Thaxton, R.E., Ganem, V.J., Zarzabal, L.A. and Bebarta, V.S., 2014. A comparison of simulation-based education versus lecture-based instruction for toxicology training in emergency medicine residents. *Journal of Medical Toxicology*, *10*(4), 364-368.

Maertens, H., Aggarwal, R., Moreels, N., Vermassen, F. and Van Herzeele, I., 2017. A proficiency based stepwise endovascular curricular training program enhances operative performance in real life: a randomised controlled trial. *European Journal of Vascular & Endovascular Surgery*, 54(3), 387-396.

Mansoory, M.S., Khazaei, M.R., Azizi, S.M. and Niromand, E., 2021. Comparison of the effectiveness of lecture instruction and virtual reality-based serious gaming instruction on the medical students’ learning outcome about approach to coma. *BMC Medical Education*, *21*(1), 1-7.

Matsumura, Y., Shinno, H., Mori, T. and Nakamura, Y., 2018. Simulating clinical psychiatry for medical students: a comprehensive clinic simulator with virtual patients and an electronic medical record system. *Academic Psychiatry*, *42*(5), 613-621.

McIntosh, K.S., Gregor, J.C. and Khanna, N.V., 2014. Computer-based virtual reality colonoscopy simulation improves patient-based colonoscopy performance. *Canadian Journal of Gastroenterology & Hepatology*, *28*(4), 203-206.

Meng, W., Yue, P., Leung, J.W., Wang, H., Wang, X., Wang, F., Zhu, K., Zhang, L., Zhu, X., Wang, Z. and Zhang, H., 2020. Impact of mechanical simulator practice on clinical ERCP performance by novice surgical trainees: a randomized controlled trial. *Endoscopy*, *52*(11), 1004-1013.

Merriman, C.D., Stayt, L.C. and Ricketts, B., 2014. Comparing the effectiveness of clinical simulation versus didactic methods to teach undergraduate adult nursing students to recognize and assess the deteriorating patient. *Clinical Simulation in Nursing*, *10*(3), e119-e127.

Pietersen, P.I., Jørgensen, R., Graumann, O., Konge, L., Skaarup, S.H., Schultz, H.H.L. and Laursen, C.B., 2021. Training thoracic ultrasound skills: a randomized controlled trial of simulation-based training versus training on healthy volunteers. *Respiration*, *100*(1), 34-43.

Qi, F., Gan, Y., Wang, S., Tie, Y., Chen, J. and Li, C., 2021. Efficacy of a virtual reality–based basic and clinical fused curriculum for clinical education on the lumbar intervertebral disc. *Neurosurgical Focus*, *51*(2), e17.

Rebolledo, B.J., Hammann-Scala, J., Leali, A. and Ranawat, A.S., 2015. Arthroscopy skills development with a surgical simulator: a comparative study in orthopaedic surgery residents. *The American Journal of Sports Medicine*, *43*(6), 1526-1529.

Ros, M., Debien, B., Cyteval, C., Molinari, N., Gatto, F. and Lonjon, N., 2020. Applying an immersive tutorial in virtual reality to learning a new technique. *Neurochirurgie*, *66*(4), 212-218.

Sarı, H.Y., Öztornacı, B.Ö., Akgül, E.A., Karakul, A., Doğan, Z. and Doğan, P., 2018. The results of simulation training in pediatric nursing students’ education. *The Journal of Pediatric Research*, *5(4)*,194-200.

Schroedl, C.J., Corbridge, T.C., Cohen, E.R., Fakhran, S.S., Schimmel, D., McGaghie, W.C. and Wayne, D.B., 2012. Use of simulation-based education to improve resident learning and patient care in the medical intensive care unit: a randomized trial. *Journal of Critical Care*, *27*(2), 219.e7-219.e13.

Smelt, J.L., Corredor, C., Edsell, M., Fletcher, N., Jahangiri, M. and Sharma, V., 2015. Simulation-based learning of transesophageal echocardiography in cardiothoracic surgical trainees: a prospective, randomized study. *The Journal of Thoracic & Cardiovascular Surgery*, *150*(1), 22-25.

Smelt, J.L., Phillips, S., Hamilton, C., Fricker, P., Spray, D., Nowell, J.L. and Jahangiri, M., 2016. Simulator teaching of cardiopulmonary bypass complications: a prospective, randomized study. *Journal of Surgical Education*, *73*(6), 1026-1031.

Solymos, O., O’Kelly, P. and Walshe, C.M., 2015. Pilot study comparing simulation-based and didactic lecture-based critical care teaching for final-year medical students. *BMC Anesthesiology*, *15*(1), 1-5.

Starodub, R., Abella, B.S., Hoyt-Brennan, A.M., Leary, M., Mancini, M.E., Chittams, J. and Riegel, B., 2020. A comparative study of video lecture versus video lecture and high-fidelity simulation for training nurses on the delivery of targeted temperature management after cardiac arrest. *International Emergency Nursing*, *49*, 100829.

Stayt, L.C., Merriman, C., Ricketts, B., Morton, S. and Simpson, T., 2015. Recognizing and managing a deteriorating patient: a randomized controlled trial investigating the effectiveness of clinical simulation in improving clinical performance in undergraduate nursing students. *Journal of Advanced Nursing*, *71*(11), 2563-2574.

Stepan, K., Zeiger, J., Hanchuk, S., Del Signore, A., Shrivastava, R., Govindaraj, S. and Iloreta, A., 2017. Immersive virtual reality as a teaching tool for neuroanatomy. *International Forum of Allergy & Rhinology*, *7*(10), 1006-1013.

Subramanian, A., Timberlake, M., Mittakanti, H., Lara, M. and Brandt, M.L., 2012. Novel educational approach for medical students: improved retention rates using interactive medical software compared with traditional lecture-based format. *Journal of Surgical Education*, *69*(4), 449-452.

Succar, T., Zebington, G., Billson, F., Byth, K., Barrie, S., McCluskey, P. and Grigg, J., 2013. The impact of the Virtual Ophthalmology Clinic on medical students’ learning: a randomised controlled trial. *Eye*, *27*(10), 1151-1157.

Tawalbeh, L.I., 2020. Effect of simulation modules on Jordanian nursing student knowledge and confidence in performing critical care skills: a randomized controlled trial. *International Journal of Africa Nursing Sciences*, *13*, 100242.

Tawfik, M.M., Fayed, A.A., Dawood, A.F., Al Mussaed, E. and Ibrahim, G.H., 2020. Simulation-based learning versus didactic lecture in teaching bronchial asthma for undergraduate medical students: a step toward improvement of clinical competencies. *Medical Science Educator*, *30*(3), 1061-1068.

Tiffen, J., Corbridge, S., Shen, B.C. and Robinson, P., 2011. Patient simulator for teaching heart and lung assessment skills to advanced practice nursing students. *Clinical Simulation in Nursing*, *7*(3), e91-e97.

Tolsgaard, M.G., Ringsted, C., Dreisler, E., Nørgaard, L.N., Petersen, J.H., Madsen, M.E., Freiesleben, N.L., Sørensen, J.L. and Tabor, A., 2015. Sustained effect of simulation‐based ultrasound training on clinical performance: a randomized trial. *Ultrasound in Obstetrics & Gynecology*, *46*(3), 312-318.

Tubaishat, A. and Tawalbeh, L.I., 2015. Effect of cardiac arrhythmia simulation on nursing students’ knowledge acquisition and retention. *Western Journal of Nursing Research*, *37*(9), 1160-1174.

Urdiales, A.I.A., Struck, G.T., Guetter, C.R., Yaegashi, C.H., Temperly, K.S., Abreu, P., Tomasich, F.S. and Campos, A.C.L., 2020. Surgical cricothyroidostomy: analysis and comparison between teaching and validation models of simulator models. *Revista do Colégio Brasileiro de Cirurgiões*, *47*, e20202522.

Wong, D.T., Mehta, A., Singh, K.P., Leong, S.M., Ooi, A., Niazi, A., You-Ten, E., Okrainec, A., Patel, R., Singh, M. and Wong, J., 2019. The effect of virtual reality bronchoscopy simulator training on performance of bronchoscopic-guided intubation in patients: a randomised controlled trial. *European Journal of Anaesthesiology*, *36*(3), 227-233.

Wu, V. and Beyea, J.A., 2017. Evaluation of a web-based module and an otoscopy simulator in teaching ear disease. *Otolaryngology–Head & Neck Surgery*, *156*(2), 272-277.

Wu, V., Sattar, J., Cheon, S. and Beyea, J.A., 2018. Ear disease knowledge and otoscopy skills transfer to real patients: a randomized controlled trial. *Journal of Surgical Education*, *75*(4), 1062-1069.

Xin, B., Chen, G., Wang, Y., Bai, G., Gao, X., Chu, J., Xiao, J. and Liu, T., 2019. The efficacy of immersive virtual reality surgical simulator training for pedicle screw placement: a randomized double-blind controlled trial. *World Neurosurgery*, *124*, e324-e330.

Yamamoto, A., Obika, M., Mandai, Y., Murakami, T., Miyoshi, T., Ino, H., Kataoka, H. and Otsuka, F., 2019. Effects on postgraduate-year-I residents of simulation-based learning compared to traditional lecture-style education led by postgraduate-year-II residents: a pilot study. *BMC Medical Education*, *19*(1), 1-10.

**Supplementary Appendix 3**

R script

#add SMDs to data using metafor

data2<-escalc(measure = "SMD", m1i = mean_score_sim, m2i = mean_score_control, sd1i = SD_sim, sd2i = SD_control, n1i = participants_sim, n2i = participants_control, data = data)

#prepare data for time lag bias - mean-centre year

data2$year <- as.vector(scale(data2$year, scale = F))

#initial model without mods for overall SMD

m1<-rma.mv(yi = yi, V = vi, method = "REML", random = (~1 | study_ID/effectsize_ID), data = data2)

#calculate heterogeneity for overall model between and within studies using dmetar

mlm.variance.distribution(m1)

#generate overall orchard plot without mods using orchaRd

orchard_plot(m1, data = data2, group = "study_ID", xlab = "SMD", transfm = "none")

#model for publication + time lag bias:

m2<-rma.mv(yi = yi, V = vi, method = "REML", mods = ~year + inv, random = (~1 | study_ID/effectsize_ID), data = data2)

#convert relevant moderators to factors

data2$HCP_ID <- as.factor(data2$HCP_ID)

data2$sim_type_ID <- as.factor(data2$sim_type_ID)

data2$instruction_ID <- as.factor(data2$instruction_ID)

data2$trial_duration <- as.factor(data2$trial_duration)

data2$outcome_ID <-as.factor(data2$outcome_ID)

#model with all moderators

m3<-rma.mv(yi = yi, V = vi, method = "REML", mods = ~year + inv + HCP_ID + sim_type_ID + instruction_ID + trial_duration + MERSQI_score + outcome_ID, random = (~1 | study_ID/effectsize_ID), data = data2)

#calculate marginal means with emmeans

emm_1<-emmprep(m3)

emmeans(emm_1, specs = "HCP_ID", df = data2$ddf, weights = "prop")
